# Efficacy and Safety of Topical 5% Cannabidiol Plus Myrcene for the Treatment of Vestibulodynia: A Multi-Centric Randomized Controlled Trial

**DOI:** 10.3390/biomedicines13102440

**Published:** 2025-10-07

**Authors:** Filippo Murina, Giuseppe Ettore, Cecilia Fochesato, Maria Grazia Castiglione, Melania Caruso, Ilenia Fonti, Valeria Savasi

**Affiliations:** 1 Lower Genital Tract Disease Unit, V. Buzzi Hospital, University of the Study of Milan, 20122 Milan, Italy; cecilia.fochesato@asst-fbf-sacco.it; 2 Obstetric and Gynecological Unit, ARNAS-Garibaldi-Nesima Hospital, 95122 Catania, Italy; gettore@arnasgaribaldi.it (G.E.); mgraziacastiglione@arnasgaribaldi.it (M.G.C.); mcaruso@arnasgaribaldi.it (M.C.); ifonti@arnasgaribaldi.it (I.F.); 3 Clinical Obstetric and Gynecological, V. Buzzi Hospital, ASST-FBF-Sacco, University of the Study of Milan, 20122 Milan, Italy; valeria.savasi@unimi.it

**Keywords:** vestibulodynia, vulvodynia, vulvar pain, dyspareunia, cannabidiol, neuropathic pain

## Abstract

**Background/Objectives:** Vestibulodynia is the prevalent form of vulvodynia, causing burning, irritation, rawness, and dyspareunia sensations. This sensory abnormality suggests sensitization to neuropathic pain. **Methods:** This was a randomized double-blind trial involving patients who applied a 5% cannabidiol gel with myrcene or a placebo to their vulvar vestibule for 60 days, assessing changes in dyspareunia, pain, and vestibular cotton swab test scores on a 0–10-point VAS scale. **Results:** This study enrolled 40 women, with 20 in the active treatment group and 20 in the placebo group. All symptoms improved in both groups, but the active treatment group showed a greater reduction in VAS scores for pain and swab tests. However, dyspareunia improved significantly more in the active treatment group. **Conclusions:** Cannabidiol’s positive effects on vestibulodynia patients can be attributed to its antinociceptive and anti-inflammatory properties. It desensitizes transient vanilloid receptor potential channels subtype 1, which are found in peripheral C-fiber nociceptors and mast cells. The results also suggest that myrcene, a terpene found in cannabis, can inhibit peripheral sensitization exerted by cannabidiol.

## 1. Introduction

Chronic vulvar discomfort, typically characterized as a burning, stinging sensation linked to allodynia and hyperalgesia, is known as vulvodynia [1]. The defining feature of vulvodynia is vulvar pain that persists for at least three months and has no clear cause, but there may be underlying variables. The prevalence rate for vulvodynia, or chronic vulvar pain, can reach 10% to 16% [1]. While vulvodynia is comparable to other common chronic pain conditions like endometriosis, it is still poorly understood, frequently misdiagnosed, or even ignored by health professionals. The effects of vulvodynia include high psychological distress, significant disruption in all aspects of sexual function, and an altered quality of life [2]. Localized provoked vulvodynia, known as vestibulodynia (VBD), represents the most prevalent form of the condition, impacting approximately 80% of cases. Women with VBD frequently report vulvar pain characterized by sensations of burning, irritation, rawness, and dyspareunia (painful or difficult intercourse) [3]. The pattern of VBD responses indicates sensory abnormalities characterized by evoked pain, such as hyperalgesia or allodynia, which suggests sensitization as a manifestation of neuropathic pain [3]. This pattern aligns with biopsy studies indicating heightened innervation of the vulvar vestibule, alongside increased subepithelial heparinase activity and cytokines associated with neuroinflammatory processes. Additionally, patients with VBD report alterations in sensitivity, implying that sensory dysregulation may play a role in the manifestation of this pain condition [4]. Additionally, the discomfort associated with VBD is consistently linked to pelvic floor muscle overactivity. The extended pattern may lead to reduced tissue perfusion, muscle overactivity dysfunction, and the formation of myofascial trigger points, causing localized or radiating pain and significant tenderness [5]. Neuropathic pain and hypertonicity represent multifactorial and complex outcomes of maladaptive neuronal plasticity. VBD is probably not a singular disease but rather a collection of disorders characterized by vestibular hypersensitivity and pelvic floor hypertonic dysfunction as common endpoints. VBD is likely the result of multiple factors, including infections, hormonal changes, allergies, genetic predispositions, and mental health conditions, which can impact patients in varying ways. The disease lacks a standard treatment, and there have been few randomized controlled trials conducted; recommendations support a multi-dimensional treatment approach. Treatment for vulvodynia typically initiates with conservative management strategies, which encompass the use of hypoallergenic soaps and lotions, the exclusive wearing of cotton undergarments, and the avoidance of tight clothing or friction in the affected area [6]. Patients typically proceed to topical therapies, such as the application of lidocaine to the affected area prior to any vaginal insertion. This therapy may provide temporary relief by reducing sensation in the affected area; however, it often leads to irritative and allergic reactions [7]. The goal of this study is to carefully observe how effective and safe the combination of cannabidiol (CBD) and myrcene is for patients with VBD. The large number of cannabinoid receptors found on skin nerve fibers and mast cells suggests that cannabinoid receptor agonists could help reduce inflammation and pain, indicating that they might have a wide range of possible medical uses [8]. The non-psychoactive version of tetrahydrocannabinol (THC), called cannabidiol (CBD), has shown strong effects in reducing pain, inflammation, and nerve-related issues without causing the high associated with THC. Researchers have demonstrated that the topical application of CBD can significantly improve pain and other disturbing sensations in patients with peripheral neuropathy [9]. Myrcene (7-methyl-3-methylene-1,6-octadiene), an acyclic monoterpene, is the most prevalent monoterpene in cannabis. In preclinical studies, administration of essential oils rich in myrcene has been found to have analgesic and anti-inflammatory properties [10]. We believe that combining CBD with myrcene in a suitable oleogel could work better together to help with symptoms related to VBD. This combination may enhance the overall therapeutic effect, leading to greater relief for patients suffering from this debilitating condition. Further research is needed to explore the optimal ratios of these compounds and to evaluate their efficacy in clinical trials.

## 2. Materials and Methods

### 2.1. Study Design

This is a randomized, double-blind, controlled trial. Ospedale dei Bambini Vittore Buzzi in Milan and ARNAS—Garibaldi in Catania were both involved in the clinical study, enrolling women with VBD who met all the criteria listed below. The study protocol was registered on http://www.isrctn.com (ISRCTN78323046) (accessed on 30 September 2025):

- Women at least 18 years of age and premenopausal (absence of menstruation for 12 months);
- Experience moderate to severe pain, a minimum of 4/10 on a numeric rating scale with respect to at least 1 of 3 parameters assessed (pain, dyspareunia, and swab test) for at least 90% of sexual intercourse attempts;
- Pain limited to the vestibule during vaginal intercourse and during activities exerting pressure on the vestibule (tampon insertion, tight jeans or pants, cycling, and horseback riding).
- Presence of VBD for at least 6 months and diagnosed according to the standardized gynecological examination protocol.

We excluded subjects according to the following criteria:

- Active vulvo-vaginal infections at the time of their gynecological examination;
- Genital bleeding of unknown origin;
- Patients concomitantly included in different interventional clinical trials;
- Unwillingness to provide informed consent to the trial;
- Women who used topical drugs in the past 30 days.

The enrolled patients received, based on random assignment, a dispensing system containing 5% CBD gel with myrcene or a placebo dispenser containing the vehicle without active ingredients (the vehicle was equipped with bio-adhesive technology). The patients were instructed to apply the gel to the vulvar vestibule (two pumps from the dispenser) once daily for 60 days before going to bed. The active formulation consists of an anhydrous oleogel, meaning that it contains no water, has no pH, and absolutely does not interfere with the pH of the vulvar area. The anhydrous formulation and the airless container used preserve the product’s integrity and also eliminate the need for preservatives or other potentially aggressive substances. The preparation has been specifically designed for vulvar use, and the applied pharmaceutical technique of microemulsification of the components allows for a uniform distribution of components.

### 2.2. Outcomes

The outcome was determined by addressing all the examined symptoms, including pain and dyspareunia, and by comparing the average changes in scores on a 0–10 point visual analog scale (VAS) in both absolute and percentage terms. The vestibular cotton swab test, which uses a small cotton-tipped stick gently rolled over different areas of the vestibule while asking the person to rate their pain from 1 (no pain) to 10 (worst pain), was also assessed.

### 2.3. Statistical Analysis

To calculate the sample size for paired differences, the ‘http://statulator.com’ (accessed on 10 June 2025) program was utilized. With a power of 80% and a level of significance of 5%, for detecting a mean of the differences of the VAS scale of 1.5 (20%) between pairs and assuming the standard deviation of the differences to be 2, we recruited 40 participants, with 20 in each group. We summarized patients’ demographic characteristics, clinical history, and baseline symptoms (i.e., pain, dyspareunia, cotton swab test), applying appropriate standard descriptive statistics depending on the type of variable. Continuous variables were expressed as mean and standard deviation or median and range as appropriate. Categorical variables were described using absolute and relative frequencies (percentages). Differences in baseline characteristics between the two comparison groups (i.e., active treatment and placebo group) were tested using the independent *t*-test or the Mann–Whitney U test for continuous variables and Fisher’s exact test for categorical variables. This allowed us to identify any significant differences in baseline characteristics between the two study groups that might affect the outcomes, and these differences should be taken into account when interpreting the results. The effectiveness of both active treatment and placebo was evaluated by comparing the outcomes at different time points (i.e., baseline and follow-up) within each group separately (within-group comparison). The *t*-test for paired data was used to determine whether there were significant differences in the VAS scores (recorded for pain, dyspareunia, and cotton swab test) between these time points within each group.

To assess the comparative effectiveness between the active treatment and placebo, a between-group comparison was performed by comparing the change in outcomes (i.e., the absolute and relative difference in VAS scores from baseline to follow-up) between the two groups. The relative difference in VAS scores was calculated as the percentage change from the baseline value as follows:

(baseline VAS—follow-up VAS)/baseline VAS×100(1)

This analysis helps determine whether one group had a greater change in VAS scores over time than the other. The *t*-test for independent data was used for this comparison.

Statistical significance was considered when *p* < 0.05 (two-tailed). Statistical analyses were conducted with SAS statistical software version 9.4 (SAS Institute, Inc., Cary, NC, USA), and graphs were created with R 4.2 software.

## 3. Results

A total of 40 women were enrolled; 20 were assigned to the active treatment group (treated with a highly bioadhesive oleogel enriched with CBD plus myrcene) and 20 to the placebo group (treated with a highly bioadhesive topical oleogel without active ingredients). The consort flow diagram for this study is shown in Figure S1.

The baseline demographic and clinical characteristics of the patients are summarized in Table 1. No differences were found between the two groups for all parameters considered. No woman has abandoned the study or complained of discomfort/adverse reactions.

**Figure 1:**
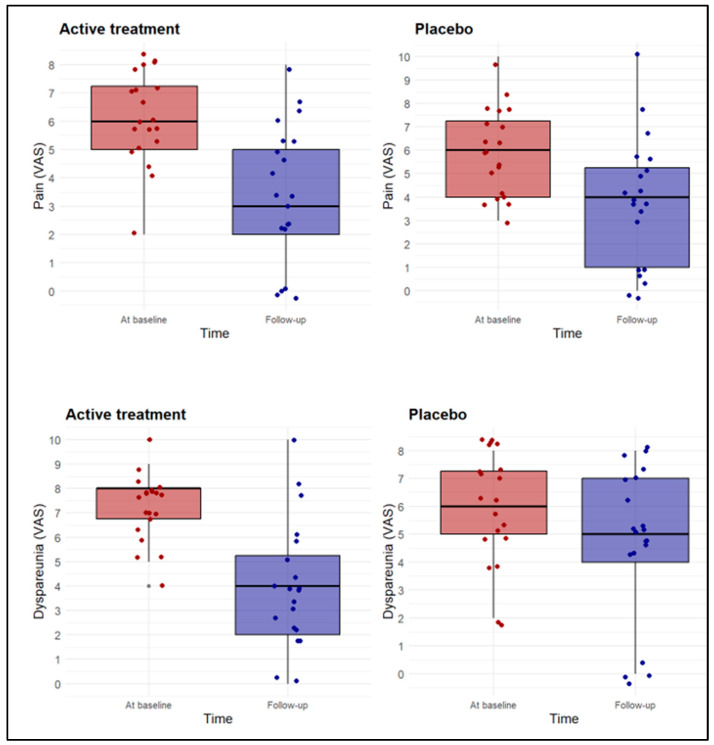
Distribution of VAS scores of symptoms at baseline and at follow-up by allocation group (active treatment and placebo). Points are randomly spaced along the x and y axes to display overlapping observations. Red box and dots = baseline evaluation; blue box and dots = follow-up evaluation. Middle line = median.

In both the active treatment and placebo groups, all symptoms, including pain, dyspareunia, and swab test results, improved, with a significant decrease in VAS scores (Table 2, Figure 1). The absolute reduction in VAS scores for pain (*p* = 0.39) and swab tests (*p* = 0.06) was greater in the active treatment group compared to the placebo group, although the statistical significance was not reached. The mean differences (MDs) for these outcomes were as follows: for pain, −2.8 (95% CI: −3.9 to −1.6) in the active treatment group and −2.1 (95% CI: −3.1 to −1.1) in the placebo group; for dyspareunia, −3.1 (95% CI: −4.4 to −1.7) in the active treatment group and −1.6 (95% CI: −2.4 to −0.8) in the placebo group. However, dyspareunia improved significantly more in the active treatment group than in the placebo group (*p* < 0.01). Specifically, the MD in the VAS score between baseline and end of treatment was a reduction of 3.3 points (95% CI: −4.4 to −2.1) in the active treatment group compared to a reduction of 1.2 points (95% CI: −1.9 to −0.5) in the placebo group. It is important to note that the active treatment group started with higher baseline dyspareunia VAS scores, which allowed for a greater absolute reduction.

**Figure 2:**
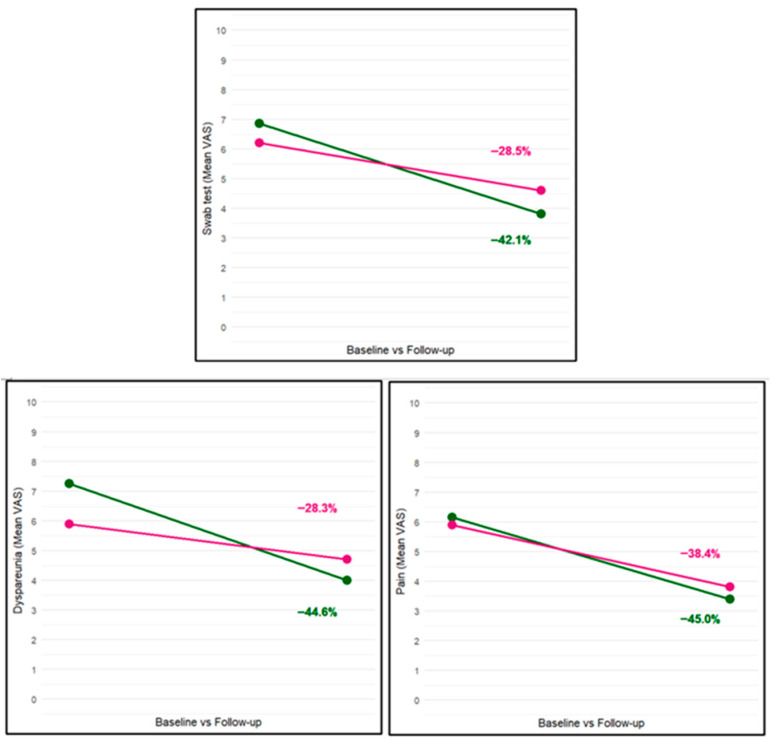
Absolute and relative difference in VAS scores of all symptoms from baseline to follow-up by allocation group (active treatment in green and placebo in pink).

Upon evaluating the percentage change from the baseline value, we found that the active treatment group experienced all changes at a higher rate than the placebo group, although statistical significance was not reached: −45.0% in the active treatment group versus −38.4% in the placebo group for pain (*p* = 0.61), −44.6% versus −28.3% for dyspareunia (*p* = 0.17), and −42.1% versus −28.5% for the cotton swab test (*p* = 0.24) (Figure 2).

## 4. Discussion

We investigated pain and dyspareunia reduction with topical CBD 5% plus myrcene as a treatment option for VBD in a multicentric randomized double blind controlled trial (RCT). We found an improvement in all the symptoms we looked at—pain, dyspareunia, and tenderness measured by touch testing at the most tender vestibular area (cotton swab test), with a higher efficacy after the treatment with topical CBD 5% plus myrcene in the active treatment group compared to the placebo group. To our knowledge, this is the first multicenter RCT to evaluate a topical CBD compound in the treatment of VBD. More specifically, we reported a reduction in all considered parameters of about 50% compared to baseline with the use of the technologically advanced formulation enriched with CBD and myrcene. There are several pieces of evidence that indicate that the vulvar vestibule in VBD has two unusual problems: an increase in sensory nerve fibers and the presence of neuroinflammatory cells, both of which are associated with nociplastic pain [11].

Nociplastic pain is an advancement in the categorization of neuropathic pain, occurring when the body’s pain perception is altered despite the absence of evident injury or disease that would typically induce discomfort [12]. In this trial, the positive effects obtained with the use of topical CBD in patients with VBD can be related to its antinociceptive and anti-inflammatory action. Cannabinoids constitute a class of biological compounds capable of binding to cannabinoid receptors (CBs). CBs of type 1 (CB1) are mainly located in the CNS, and their distribution indicates a crucial function in regulating motor activities, cognition, and memory. Conversely, CBs of type 2 (CB2) are primarily located in the peripheral nerve terminals and immune cells [13].

CBD exhibits a very low agonistic affinity for CB1, which could also explain the lack of psychotropic activity associated with this compound, while the analgesic and anti-inflammatory response is most likely associated with CB2 activation [14]. Furthermore, CBD may mainly exert its antinociceptive effect via the desensitization of transient vanilloid receptor potential channels subtype 1 (TRPV1), as was shown in preclinical studies [15]. TRPV1 channels are polymodal receptors predominantly located in peripheral C-fiber nociceptors, but they can also be found in several non-neuronal cells (e.g., granulocytes, lymphocytes, mast cells) [16]. Available data suggest that TRPV1 plays an important role in the pathophysiology of neuropathic pain. Furthermore, researchers have shown that an imbalance in pro-resolving lipids, along with heightened responses to inflammatory stimuli in the vulvar vestibule, promotes TRPV activation [17]. Topical CBD has demonstrated the ability to reduce proinflammatory cytokine levels, such as IL-6 and IL-17, while pretreatment with CBD has led to an increase in IL-10, an anti-inflammatory cytokine [18]. Evidence indicates that cannabinoids can interact with multiple spinal and supraspinal sites to increase nociceptive thresholds. Numerous studies demonstrate that both endogenous and exogenous cannabinoids influence pain transmission at the spinal level through specific CB1 receptors, with significant peripheral effects also observed. Cannabinoids may increase nociceptive thresholds through the inhibition of the cAMP’s second messenger system in peripheral tissues. Furthermore, reports indicate that CB1 receptor agonists suppress the release of substance P and calcitonin gene-related peptides (CGRPs) from primary afferent neurons [19].

Although there are no studies regarding the topical use of CBD in vulvodynia, in an RCT testing topical CBD oil in 29 patients with peripheral neuropathic pain compared to the placebo, CBD showed a more pronounced pain-reducing effect than the placebo [20]. We used myrcene in association with CBD in our RCT. The cannabis plant contains a vast array of chemical constituents, some of which have been shown to possess antinociception or anti-hyperalgesic effects, including 120 identified terpenes, and myrcene is 1 of the 8 predominant terpenes. Preclinical rodent studies have reported that myrcene possesses antinociceptive effects in various nociceptive assays through the engagement of CB1. We can also attribute our results to the positive synergistic effect of myrcene on the inhibition of peripheral sensitization exerted by CBD. Localized signs and symptoms of neuropathic pain (e.g., allodynia, hyperalgesia) can be a major target for analgesics applied topically. Provoked VBD is the pattern more involved in the definition of “localized neuropathic pain” (LNP), in which a topical treatment should be considered [21], and this is the reason why we have chosen a topical compound with CBD plus myrcene. LNP is a form of pain defined by a consistent and localized area of maximum intensity accompanied by altered sensory signs and symptoms, such as allodynia and hyperalgesia, which are indicative of neuropathic or nociplastic pain, and is perceived at a superficial level. The proposed definition of LNP may assist clinicians in optimally selecting patients who are the most suitable candidates for topical treatments. The routes through which topical CBD can be administered are pivotal, as well as the CBD delivery systems and excipients. The formulation used in our study is an oleogel with high bioadhesiveness, where the texture itself can bring benefits to the vestibular area, with a greater persistence of the active ingredient, in addition to a protective and soothing action on the vestibular mucosa. These elements can explain the partial positive effects on the symptoms observed in the placebo group. Furthermore, this application method is of great interest because it avoids the problems associated with conventional modes of administration and can procure high bioavailability. The penetration of therapeutic agents is also enhanced by the absence of the stratum corneum in the vestibular mucosa, the outer layer of the epidermis that is one of the limiting steps for topical and transdermal applications. The small sample size represents a limit of this study, not providing enough statistical power to detect a significant difference in the between-group comparison.

A further limitation includes the brief follow-up period, the subjectivity of self-reported outcomes, and the variability among participants. Our findings suggest further applications of this formulation, such as relieving discomfort, burning, and itching associated with various vulvar and vaginal conditions. This includes symptoms linked to vulvovaginal atrophy (such as during menopause); microtraumas from activities like intercourse, physical exertion, or hair removal; and irritation caused by conditions such as contact dermatitis, infections, and vulvar dermatoses.

## 5. Conclusions

Research studies conclude that a multimodal approach is essential for diagnosing and treating women with persistent vulvar pain, addressing both biological and psychological contributing factors. Further research with prolonged follow-up and implementation of different multimodal treatments is needed. The results indicate that the combination of topical CBD and myrcene is effective in alleviating pain and addressing sexual issues in patients with VBD. We recommend that this approach be administered not as a standalone therapy but as an important aspect of multimodal therapy to enhance its effectiveness in treating VBD.

## Figures and Tables

**Table 1:** The baseline demographic and clinical characteristics of the patients.

Parameter	Active Treatment *n* = 20	Placebo *n* = 20	*p*-Value
Age (years)	27.5 (20.0–49.0)	29.5 (22.0–49.0)	0.39
Duration of the disease (months)	22.0 (6.0–48.0)	24.0 (6.0–120.0)	0.61
Current use of hormonal contraceptive	4 (20.0)	6 (30.0)	0.72
Burning/pain (VAS)	6.1 ± 1.6	5.9 ± 1.9	0.65
Dyspareunia (VAS)	7.3 ± 1.4	5.9 ± 1.9	0.02
Cotton swab test (VAS)	6.9 ± 1.6	6.2 ± 1.7	0.22

Continuous data are expressed as median (range), and categorical data are expressed as absolute numbers (percentages). Abbreviations: VAS, visual analogue score. Data are expressed as mean ± standard deviation.

**Table 2:** Symptoms at baseline and follow-up in active treatment and placebo groups.

Parameter	Active Treatment *n* = 20			Placebo *n* = 20		
	Baseline	Follow-up	*p*-value	Baseline	Follow-up	*p*-value
Burning/pain (VAS)	6.1 ± 1.6	3.4 ± 2.4	<0.01	5.9 ± 1.9	3.8 ± 2.8	<0.01
Dyspareunia (VAS)	7.3 ± 1.4	4.0 ± 2.6	<0.01	5.9 ± 1.9	4.7 ± 2.7	<0.01
Cotton swab test (VAS)	6.9 ± 1.6	3.8 ± 2.4	<0.01	6.2 ± 1.7	4.6 ± 2.4	<0.01

Continuous data are expressed as median (range), and categorical data are expressed as absolute numbers (percentages). Abbreviations: VAS, visual analogue score. Data are expressed as mean ± standard deviation.

## Data Availability

The original contributions presented in this study are included in the article/supplementary material. Further inquiries can be directed to the corresponding author.

## Supplementary Materials

The following supporting information can be downloaded at https://www.mdpi.com/article/10.3390/biomedicines13102440/s1. Figure S1: Consort flow diagram.

## Abbreviations

The following abbreviations are used in this manuscript:

VBD	Vestibulodynia;
THC	Tetrahydrocannabinol;
CBD	Cannabidiol;
VAS	Visual analogue scale;
MD	Mean difference;
CBs	Cannabinoid receptors;
TRPV1	Transient vanilloid receptor potential channels subtype 1;
RCT	Randomized controlled trial;
LNP	Localized neuropathic pain.
