# Supplementary figures and images for: Efficacy and Safety of Topical 5% Cannabidiol Plus Myrcene for the Treatment of Vestibulodynia: A Multi-Centric Randomized Controlled Trial
Source: Biomedicines. 2025 Oct 7;13(10):2440. doi: 10.3390/biomedicines13102440 (PMC12561628; doi:10.3390/biomedicines13102440)

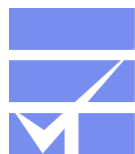

# CONSORT

TRANSPARENT REPORTING of TRIALS

## CONSORT Flow Diagram

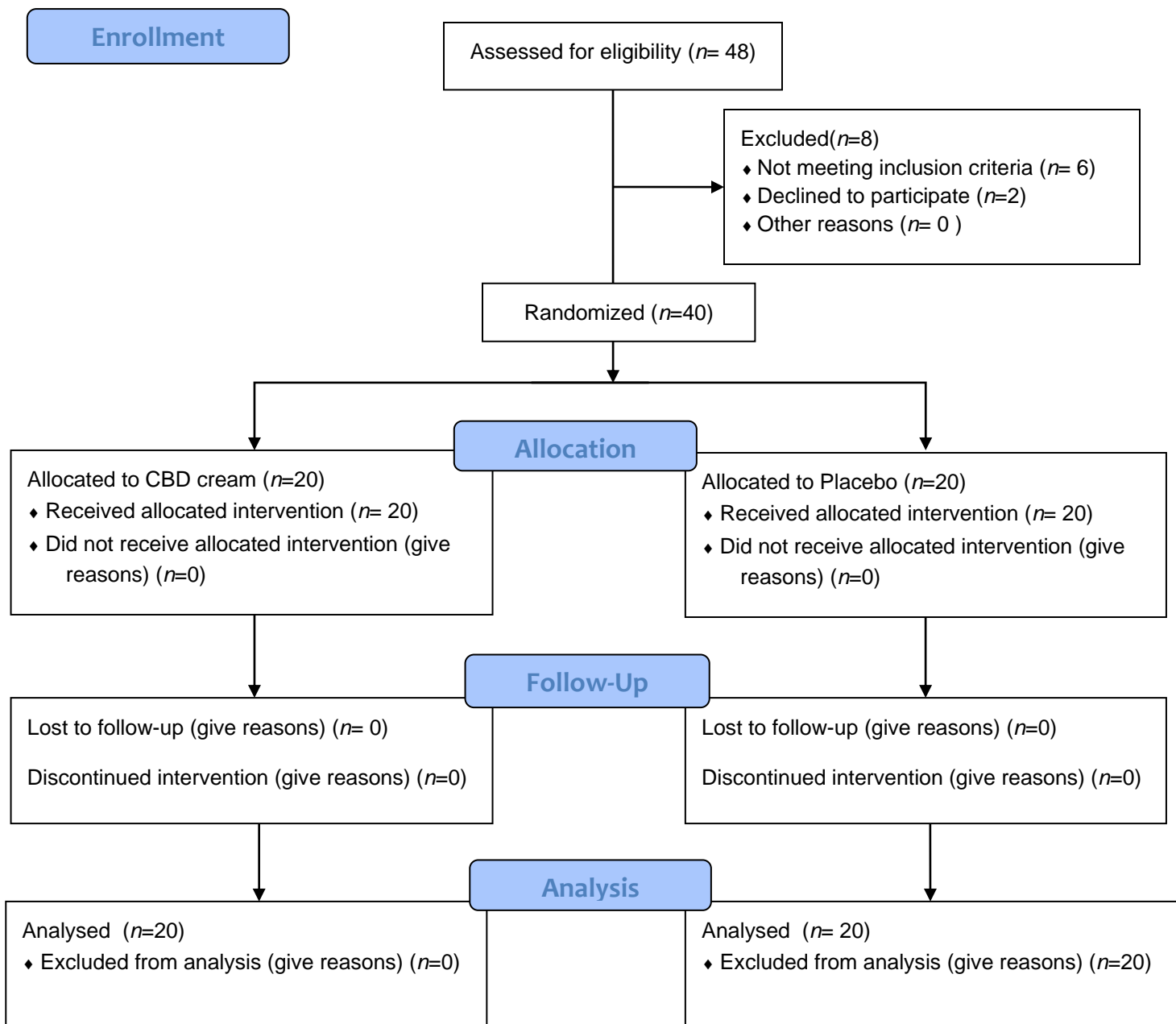

Supplement: Supplementary file 1 [file biomedicines-13-02440-s001.zip › biomedicines-3798996-supplementary.pdf]
